# Supplementary material for: Investigation of the Pharmacodynamic Components of Gastrodia elata Blume for Treatment of Type 2 Diabetes Mellitus through HPLC, Bioactivity, Network Pharmacology and Molecular Docking
Source: Int J Mol Sci. 2024 Sep 29;25(19):10498. doi: 10.3390/ijms251910498 (PMC11476761; doi:10.3390/ijms251910498)
Supplement: Supplementary file 1 [file ijms-25-10498-s001.zip › ijms-3193492-supplementary.pdf]

Investigation of the Pharmacodynamic Components of *Gastrodia elata* Blume for Treatment of Type 2 Diabetes Mellitus through HPLC, Bioactivity, Network Pharmacology and Molecular Docking

Xiu Yang[1,2,3,†], Lilang Li[1,2,†], Yanfang Yan[1,2], Qiji Li[1,2], Liangqun Li[1,2], Lishou Yang[1,2], Mei Peng[1,2], Juan Yang[1,2], Xiaosheng Yang[1,2,*], and Ming Gao[1,2,*]

[1] State Key Laboratory for Functions and Applications of Medicinal Plants, Guizhou Medical University, Guiyang, 550014, PR China

[2] Natural Products Research Center of Guizhou Province, Guiyang, 550014, PR China

[3] College of pharmacy, Guizhou University of Traditional Chinese Medicine, Guiyang 550014, China

*Correspondence: gaoming@gmc.edu.cn (M. Gao), Tel.: +86-18345701431; yangxiaosheng@gmc.edu.cn (X. Yang), Tel.: +86-13595099817

†These authors contributed equally to this work.

Contents:

Table S**1**: The result of Precision, Stability, Repeatability, and Accuracy of HPLC…………………………………..……………………....3

Table S**2**: The CV and RSD results of the GE samples………………..…4

Table S**3**: Target genes of GE Bioactive Compound……………………..4

Table S**4**: The 285 potential common targets of GE marker components and T2DM……………………………………………………………..10

Table S**5**: The degree value of nodes in protein–protein interaction network………………………………………………………………17

Figure S**1**: The calibration range and calibration curve equations for compounds……………………….……………………………...18

Figure S**2**: The content difference of marker components in GE of batches plots………………………..……………………………….....19

supplementary information: Precision, Stability, Repeatability, and Accuracy procedure.

The precision was evaluated according to the assay of DJW-6, in which the solution was analyzed for six times in a day, to evaluate the intra-day precision, and was analyzed on three consecutive days, to evaluate the inter-day precision. The stability was tested with the DJW-6 solution that was stored at room temperature (25 ± 5 ◦C) and analyzed at 0, 2, 4, 6, 8, 12, and 24 h. In the repeatability test, six duplicates of DFW-3 were extracted and analyzed, according to the sample preparation procedure, and the HPLC method. The accuracy of the method is determined by adding a known amount of a standard at the time of GE sample extraction.

The GAS, HBA, parishin E, parishin B, parishin C and parishin A were accurately weighed with the amount of 2.1, 23.05, 17.75, 5.75, 26.75 and 10.25 mg and placed in a 5 mL brown measuring bottle, respectively. Then, 60% methanol was used to set the volume to the scale to get a mixed control solution. The GE sample (DFW-3, 1 g) was precisely weighted and add 1 mL of the above mixed control solution, and then prepare six parallel sample solutions by the same sample preparation method. The HPLC chromatographic conditions were applied with the same method on section 2.2.3. The RSD of GAS, HBA, parishin E, parishin B, parishin C and parishin A chromatographic peaks were calculated based on the HPLC results. Recovery rate was used as the evaluation index and calculated as Recovery rate (%) = (Found amount − Known amount) × 100%/Added amount.

Table S1: The result of Precision, Stability, Repeatability, and Accuracy of HPLC.

Compound	Precision RSD (%)		Stability	Repeatability	Sample accuracy	
	intra-day	inter-day	RSD (%)	RSD (%)	Recovery (%)	RSD (%)
Gastrodin	1.88	1.07	1.87	0.67	98.03	1.70
p-Hydroxy benzyl alcohol	0.79	1.64	1.59	1.84	99.22	1.50
Parishin E	0.82	1.18	0.97	1.36	103.15	1.69
Parishin B	1.82	0.58	1.04	1.91	101.93	1.73
Parishin C	0.83	2.53	0.95	1.42	96.98	1.73
Parishin A	0.44	0.95	0.73	1.77	97.08	1.93

Table S2: The CV and RSD result of the GE samples.

	Peak Area of Six Characteristic Peaks					
	**GAS**	**HBA**	**Parishin E**	**Parishin B**	**Parishin C**	**Parishin A**
**intra-day Precision**	2046	702	1980	3188	561	9485
	1939	696	1987	3133	558	9582
	1987	705	1967	3074	554	9560
	1994	701	1962	3181	548	9501
	2031	690	2008	3079	557	9480
	2006	695	1980	3058	559	9529
C.V.	1.88%	0.79%	0.82%	1.82%	0.83%	0.44%
RSD	1.88%	0.79%	0.82%	1.82%	0.83%	0.44%
**inter-day Precision**						
Day 1	1913	759	2003	3117	549	9593
	1906	761	1984	3122	551	9505
Day 2	1902	730	1953	3153	575	9452
	1935	749	2012	3127	562	9649
Day 3	1957	743	1987	3125	556	9429
	1923	737	1959	3161	585	9613
C.V.	1.07%	1.64%	1.18%	0.58%	2.53%	0.95%
RSD	1.07%	1.64%	1.18%	0.58%	2.53%	0.95%
**Stability**	1963	707	2001	3079	546	9421
	1973	687	1984	3161	556	9555
	1969	692	1980	3133	548	9582
	1975	682	1953	3135	542	9449
	2046	696	1951	3098	548	9529
	2037	710	1975	3087	542	9433
C.V.	1.87%	1.59%	0.97%	1.04%	0.95%	0.73%
RSD	1.87%	1.59%	0.97%	1.04%	0.95%	0.73%
**Repeatability**	1983	714	2021	3034	554	9688
	1993	687	1974	3189	549	9575
	1985	691	1980	3153	553	9372
	1987	682	1953	3135	542	9698
	2013	696	2013	3088	536	9339
	2011	710	1965	3059	538	9363
C.V.	0.67%	1.84%	1.36%	1.91%	1.42%	1.77%
RSD	0.67%	1.84%	1.36%	1.91%	1.42%	1.77%
**Accuracy**	523	4292	1934	971	1298	6357
	535	4261	2008	976	1247	6492
	541	4268	2013	1012	1263	6248
	528	4344	1938	958	1262	6184
	519	4258	2041	962	1248	6411
	538	4398	2064	1007	1259	6308
DFW-3 (2g)	524	4291	1935	972	1296	6359
**Recovery**	98.03%	99.22%	103.15%	101.93%	96.98%	97.08%
C.V.	1.61%	1.31%	2.65%	2.34%	1.41%	1.76%
RSD	1.61%	1.31%	2.65%	2.34%	1.41%	1.76%

C.V. (%) = $\delta/\mu \times 100$, δ—The standard deviation of peak area and μ—The average value of each

Table S3: Target genes of GE Bioactive Compound.

NO.	Target	Common name
77	Serine/threonine-protein kinase/endoribonuclease IRE1	ERN1
78	Endothelin receptor ET-A (by homology)	EDNRA
79	Cystic fibrosis transmembrane conductance regulator	CFTR

80	Caspase-6	CASP6
81	Caspase-7	CASP7
82	Caspase-8	CASP8
83	Endothelin-converting enzyme 1	ECE1
84	Caspase-1	CASP1
85	Caspase-2	CASP2
86	Matrix metalloproteinase 16	MMP16
87	Alpha-ketoglutarate-dependent dioxygenase FTO	FTO
88	Adenosine A2b receptor	ADORA2B
89	Matrix metalloproteinase 9	MMP9
90	Matrix metalloproteinase 14	MMP14
91	Dopamine D2 receptor	DRD2
92	Dopamine D4 receptor	DRD4
93	G-protein coupled receptor 35	GPR35
94	Thymidine phosphorylase	TYMP
95	Acyl-CoA: dihydroxyacetone phosphate acyltransferase	GNPAT
96	Phospholipase A2 group 1VB	PLA2G4B
97	Inosine-5'-monophosphate dehydrogenase 1	IMPDH1
98	Inosine-5'-monophosphate dehydrogenase 2	IMPDH2
99	Epidermal growth factor receptor erbB1	EGFR
100	Acetylcholinesterase	ACHE
101	Carbonic anhydrase IV	CA4
102	Carbonic anhydrase XIII (by homology)	CA13
103	Carbonic anhydrase VB	CA5B
104	Serotonin 3a (5-HT3a) receptor	HTR3A
105	Carbonic anhydrase VII	CA7
106	Alpha-(1,3)-fucosyltransferase 7	FUT7
107	PI3-kinase p110-alpha/p85-alpha	PIK3CA
108	Arachidonate 5-lipoxygenase	ALOX5
109	Nitric oxide synthase, inducible	NOS2
110	Adenosine A2a receptor	ADORA2A
111	Neuromedin-U receptor 2	NMUR2
112	Gamma-butyrobetaine dioxygenase	BBOX1
113	Estrogen receptor alpha	ESR1
114	GABA-A receptor; gamma-2	GABRA2
115	Tyrosinase	TYR
116	Glutathione reductase	GSR
117	Alpha-2a adrenergic receptor	ADRA2A
118	Adrenergic receptor alpha-2	ADRA2C
119	Alpha-2b adrenergic receptor (by homology)	ADRA2B
120	Adrenergic receptor beta	ADRB2
121	Alpha-1a adrenergic receptor	ADRA1A
122	Inhibitor of apoptosis protein 3	XIAP
123	Thymidine kinase, cytosolic	TK1

124	Chymase	CMA1
125	Telomerase reverse transcriptase	TERT
126	Serotonin 2b (5-HT2b) receptor	HTR2B
127	GABA-A receptor; alpha-1	GABRA1
128	Serotonin 2c (5-HT2c) receptor	HTR2C
129	Estradiol 17-beta-dehydrogenase 2	HSD17B2
130	Estradiol 17-beta-dehydrogenase 1	HSD17B1
131	Acyl coenzyme A: cholesterol acyltransferase	CES1
132	Carboxylesterase 2	CES2
133	Inhibitor of NF-kappa-B kinase (IKK)	CHUK
134	Epoxide hydrolase 1 (by homology)	EPHX1
135	7,8-dihydro-8-oxoguanine triphosphatase	NUDT1
136	Estrogen-related receptor gamma	ESRRG
137	Transient receptor potential cation channel subfamily A member 1	TRPA1
138	Myeloperoxidase	MPO
139	Matrix metalloproteinase 2	MMP2
140	Nitric-oxide synthase, brain	NOS1
141	Nitric-oxide synthase, endothelial	NOS3
142	Monoamine oxidase B	MAOB
143	Guanine deaminase	GDA
144	Serotonin 2a (5-HT2a) receptor (by homology)	HTR2A
145	Glutathione S-transferase Pi	GSTP1
146	Methionine aminopeptidase 2	METAP2
147	Glutathione S-transferase Mu 2	GSTM2
148	Prostatic acid phosphatase	ACPP
149	Leukocyte common antigen	PTPRC
150	Steroid 5-alpha-reductase 2	SRD5A2
151	Glycine receptor subunit alpha-2 (by homology)	GLRA2
152	Pyruvate kinase isozymes M1/M2	PKM
153	Glucocorticoid receptor	NR3C1
154	Progesterone receptor	PGR
155	Estradiol 17-beta-dehydrogenase 3	HSD17B3
156	Bromodomain-containing protein 4	BRD4
157	cAMP-dependent protein kinase alpha-catalytic subunit	PRKACA
158	Serine/threonine-protein kinase AKT	AKT1
159	Protein tyrosine kinase 2 beta	PTK2B
160	Histamine H3 receptor	HRH3
161	Histamine H4 receptor	HRH4
162	Androgen Receptor (by homology)	AR
163	Cathepsin G	CTSG
164	L-lactate dehydrogenase A chain	LDHA
165	L-lactate dehydrogenase B chain	LDHB
166	Thymidine phosphorylase (by homology)	TYMP

167	Alpha-1d adrenergic receptor	ADRA1D
168	Mu opioid receptor (by homology)	OPRM1
169	Squalene monooxygenase (by homology)	SQLE
170	HMG-CoA reductase (by homology)	HMGCR
171	Neuronal acetylcholine receptor protein alpha-4 subunit	CHRNA4
172	Neuronal acetylcholine receptor; alpha4/beta2	CHRNA4<br>CHRNB2
173	Neuronal acetylcholine receptor; alpha3	CHRNA3
174	Aldose reductase (by homology)	AKR1B1
175	Pyrimidinergic receptor P2Y4	P2RY4
176	Purinergic receptor P2Y2	P2RY2
177	Pyrimidinergic receptor P2Y6	P2RY6
178	Eukaryotic initiation factor 4A-I	EIF4A1
179	Heat shock protein HSP 90-alpha	HSP90AA1
180	T-cell protein-tyrosine phosphatase	PTPN2
181	Phospholipase C-delta-1	PLCD1
182	Inositol 1,4,5-trisphosphate receptor type 1	ITPR1
183	Inositol 1,4,5-trisphosphate receptor	ITPR3
184	GAR transformylase	GART
185	Thrombin and coagulation factor X	F10
186	AMY1C	AMY1A
187	Plasminogen activator inhibitor-1	SERPINE1
188	Beta-secretase 1	BACE1
189	Platelet activating factor receptor	PTAFR
190	Thymidylate synthase (by homology)	TYMS
191	Galectin-3	LGALS3
192	Galectin-9	LGALS9
193	Norepinephrine transporter	SLC6A2
194	2'-deoxynucleoside 5'-phosphate N-hydrolase 1	DNPH1
195	RNase L (by homology)	RNASEL
196	Folylpoly-gamma-glutamate synthetase	FPGS
197	Integrin alpha-IIb	ITGA2B
198	Dihydrofolate reductase	DHFR
199	Vascular endothelial growth factor A	VEGFA
200	Acidic fibroblast growth factor	FGF1
201	Basic fibroblast growth factor	FGF2
202	Heparanase	HPSE
203	Adenylosuccinate synthetase 2	ADSS
204	Folate receptor alpha	FOLR1
205	Purinergic receptor P2Y1	P2RY1
206	Somatostatin receptor 5	SSTR5
207	Somatostatin receptor 2	SSTR2
208	Somatostatin receptor 4	SSTR4
209	Somatostatin receptor 1	SSTR1

210	Somatostatin receptor 3	SSTR3
211	Purinergic receptor P2Y12	P2RY12
212	Protein kinase C delta	PRKCD
213	Protein kinase C gamma	PRKCG
214	Protein kinase C alpha	PRKCA
215	Protein kinase C beta	PRKCB
216	Protein kinase C epsilon	PRKCE
217	Protein kinase C eta	PRKCH
218	Protein kinase C theta	PRKCQ
219	Glutaminyl-tRNA synthetase	QARS
220	AICAR transformylase	ATIC
221	Ectonucleotide pyrophosphatase	ENPP1
222	Free fatty acid receptor 1	FFAR1
223	Leukocyte elastase	ELANE
224	Solute carrier family 28member 3	SLC28A3
225	Ileal bile acid transporter	SLC10A2
226	CD22	CD22
227	Cyclooxygenase-2	PTGS2
228	Induced myeloid leukemia cell differentiation protein Mcl-1	MCL1
229	Butyrylcholinesterase	BCHE
230	Interleukin-2	IL2
231	P-glycoprotein 1	ABCB1
232	Aldehyde dehydrogenase	ALDH2
233	Niemann-Pick C1-like protein 1	NPC1L1
234	Metabotropic glutamate receptor 2 (by homology)	GRM2
235	Carbonic anhydrase XIII	CA13
236	Cytosolic phospholipase A2	PLA2G4A
237	TNF-alpha	TNF
238	Glucagon receptor	GCGR
239	Alpha-1b adrenergic receptor	ADRA1B
240	C-C chemokine receptor type 6	CCR6
241	Vanilloid receptor (by homology)	TRPV1
242	Peroxisome proliferator-activated receptor gamma	PPARG
243	Peroxisome proliferator-activated receptor alpha	PPARA
244	Peroxisome proliferator-activated receptor delta	PPARD
245	Acetyl-CoA carboxylase 2	ACACB
246	Kappa Opioid receptor	OPRK1
247	Troponin, cardiac muscle	TNNC1
248	Squalene synthetase (by homology)	FDFT1
249	Histone acetyltransferase p300	EP300
250	Thymidylate synthase	TYMS
251	Protein phosphatase 2C alpha	PPM1A
252	Solute carrier family 22member 2	SLC22A2
253	Multidrug and toxin extrusion protein 1	SLC47A1

254	Plasminogen	PLG
255	Plasma kallikrein	KLKB1
256	Thrombin	F2
257	Urokinase-type plasminogen activator	PLAU
258	Leukotriene A4 hydrolase	LTA4H
259	Lymphocyte differentiation antigen CD38	CD38
260	Monoamine oxidase A	MAOA
261	Cannabinoid receptor 2	CNR2
262	RNase L	RNASEL
263	Thromboxane-A synthase	TBXAS1
264	Integrin alpha-V/beta-3	ITGAV ITGB3
265	Integrin alpha-V/beta-6	ITGAV ITGB6
266	GAR transformylase (by homology)	GART
267	DNA (cytosine-5)-methyltransferase 3B	DNMT3B
268	Growth factor receptor-bound protein 2	GRB2
269	Integrin alpha-5/beta-1	ITGB1 ITGA5
270	Integrin alpha-4/beta-1	ITGB1 ITGA4
271	Prostanoid EP2 receptor (by homology)	PTGER2
272	Prostanoid EP4 receptor (by homology)	PTGER4
273	Peptidyl-prolyl cis-trans isomerase NIMA-interacting 1	PIN1
274	Serine palmitoyltransferase 1	SPTLC1
275	Serine palmitoyltransferase 2	SPTLC2
276	NAD-dependent deacetylase sirtuin 2	SIRT2
277	NAD-dependent deacetylase sirtuin 1	SIRT1
278	Hematopoietic cell protein-tyrosine phosphatase 70Z-PEP	PTPN22
279	Angiotensin II receptor	AGTR2
280	Cathepsin D	CTSD
281	Signal transducer and activator of transcription 6	STAT6
282	Tyrosine-protein kinase SRC	SRC
283	Trypsin III (by homology)	PRSS3
284	Trypsin I (by homology)	PRSS1
285	Cyclin-dependent kinase 5	CDK5R1
286	DNA (cytosine-5)-methyltransferase 1	DNMT1
287	Choline acetylase	CHAT
288	Breast cancer type 1 susceptibility protein	BRCA1
289	Cholecystokinin B receptor	CCKBR
290	Tyrosine-protein kinase FYN	FYN
291	Hepatic lipase	LIPC
292	Tyrosine-protein kinase LCK	LCK
293	Endothelial lipase	LIPG
294	HLA class I histocompatibility antigen A-3	HLA-A
295	Ribonucleoside-diphosphate reductase M1 chain (by homology)	RRM1
296	Leukotriene B4 receptor 1	LTB4R

297	Dual specificity phosphatase Cdc25B	CDC25B
298	Delta opioid receptor	OPRD1
299	11-beta-hydroxysteroid dehydrogenase 2	HSD11B2
300	11-beta-hydroxysteroid dehydrogenase 1	HSD11B1
301	Vascular endothelial growth factor receptor 2	KDR
302	P47929	LGALS7
303	Sodium/potassium-transporting ATPase alpha-1 chain	ATP1A1
304	Neurokinin 2 receptor	TACR2
305	3-phosphoinositide dependent protein kinase-1	PDPK1
306	Glutaminase kidney isoform, mitochondrial	GLS
307	Tyrosine-protein kinase receptor FLT3	FLT3
308	Apoptosis regulator Bcl-2	BCL2
309	Histone deacetylase 6	HDAC6
310	Histone deacetylase 8	HDAC8
311	Histone deacetylase 1	HDAC1
312	Receptor-type tyrosine-protein phosphatase alpha	PTPRA
313	Neurokinin 1 receptor (by homology)	TACR1
314	Membrane-associated guanylate kinase-related 3	MAGI3
315	Renin	REN
316	Neuropeptide Y receptor type 1	NPY1R
317	Caspase-6	CASP6
318	Caspase-7	CASP7
319	Caspase-8	CASP8
320	Caspase-2	CASP2

Table S4: The 285 potential common targets of GE marker components and T2DM.

NO.	target
1	CDK2
2	CDK1
3	ABCB1
4	ACACB
5	ACHE
6	ADA
7	ADK
8	ADORA1
9	ADORA2A
10	ADORA2B
11	ADORA3
12	ADRA1A
13	ADRA1B
14	ADRA1D
15	ADRA2A
16	ADRA2B

17	ADRA2C
18	ADRB2
19	AGTR2
20	AHCY
21	AKR1B1
22	AKR1C3
23	AKT1
24	ALDH2
25	ALOX5
26	AMY1A
27	AMY2A
28	AR
29	ATIC
30	ATP1A1
31	BACE1
32	BBOX1
33	BCHE
34	BCL2
35	BRCA1
36	BRD4
37	CA1
38	CA14
39	CA2
40	CA3
41	CA4
42	CA6
43	CA7
44	CA9
45	CASP1
46	CASP2
47	CASP3
48	CASP7
49	CASP8
50	CCKBR
51	CCR6
52	CD22
53	CD38
54	CDA
55	CDC25B
56	CDK5R1
57	CES1
58	CES2
59	CFTR
60	CHAT

61	CHUK
62	CMA1
63	CNR2
64	CTSD
65	CTSG
66	CYP19A1
67	DHFR
68	DNMT1
69	DNMT3B
70	DPP4
71	DRD2
72	DRD4
73	ECE1
74	EDNRA
75	EGFR
76	EIF4A1
77	ELANE
78	ENPEP
79	ENPP1
80	EP300
81	EPHX1
82	EPHX2
83	ERN1
84	ESR1
85	ESRRG
86	F10
87	F2
88	FBP1
89	FDFT1
90	FFAR1
91	FGF1
92	FGF2
93	FLT3
94	FOLH1
95	FOLR1
96	FTO
97	FUCA1
98	FYN
99	GABRA1
100	GABRB3
101	GAPDH
102	GART
103	GCGR
104	GLS

105	GNPAT
106	GPR35
107	GRB2
108	GRIK1
109	GRIK2
110	GRK1
111	GRM2
112	GSK3B
113	GSR
114	GSTM2
115	GSTP1
116	HDAC1
117	HDAC6
118	HDAC8
119	HK1
120	HK2
121	HLA-A
122	HMGCR
123	HPRT1
124	HPSE
125	HRH4
126	HSD11B1
127	HSD11B2
128	HSD17B1
129	HSP90AA1
130	HSPA5
131	HSPA8
132	HTR2A
133	HTR2B
134	HTR2C
135	HTR3A
136	IDO1
137	IGF1R
138	IGFBP3
139	IL2
140	IMPDH1
141	IMPDH2
142	INSR
143	ITGA2B
144	ITGAV
145	ITGB1
146	ITPR1
147	ITPR3
148	KDR

149	KLKB1
150	LCK
151	LDHA
152	LDHB
153	LGALS3
154	LGALS7
155	LGALS9
156	LIPC
157	LIPG
158	LTA4H
159	LTB4R
160	MAGI3
161	MAOA
162	MAOB
163	MAPK1
164	MCL1
165	MGAM
166	MME
167	MMP1
168	MMP13
169	MMP14
170	MMP16
171	MMP2
172	MMP7
173	MMP8
174	MMP9
175	MPO
176	NOS1
177	NOS2
178	NOS3
179	NPC1L1
180	NPY1R
181	NR3C1
182	NUDT1
183	OGA
184	OPRK1
185	OPRM1
186	P2RY1
187	P2RY12
188	P2RY2
189	P2RY4
190	PDPK1
191	PGR
192	PIK3CA

193	PIK3CG
194	PIN1
195	PKM
196	PLA2G4A
197	PLA2G4B
198	PLAU
199	PLCD1
200	PLG
201	PNP
202	PPARA
203	PPARD
204	PPARG
205	PPM1A
206	PRKACA
207	PRKCA
208	PRKCB
209	PRKCD
210	PRKCE
211	PRKCG
212	PRKCH
213	PRKCQ
214	PRSS1
215	PRSS3
216	PTAFR
217	PTGER2
218	PTGER4
219	PTGS1
220	PTGS2
221	PTK2B
222	PTPN1
223	PTPN11
224	PTPN2
225	PTPN22
226	PTPRC
227	PYGL
228	PYGM
229	REN
230	RNASEL
231	RRM1
232	SELE
233	SELL
234	SELP
235	SERPINE1
236	SI

237	SIRT1
238	SIRT2
239	SLC10A2
240	SLC22A2
241	SLC28A3
242	SLC29A1
243	SLC47A1
244	SLC5A1
245	SLC5A2
246	SLC5A4
247	SLC6A2
248	SPTLC1
249	SPTLC2
250	SQLE
251	SRC
252	SRD5A1
253	SRD5A2
254	SSTR1
255	SSTR2
256	SSTR3
257	SSTR4
258	SSTR5
259	STAT6
260	TACR1
261	TBXAS1
262	TDP1
263	TERT
264	TK1
265	TNF
266	TREH
267	TRPA1
268	TRPV1
269	TYMP
270	TYMS
271	TYR
272	VEGFA
273	XIAP
274	CCNB1
275	CDK5
276	CHRNB2
277	CHRNB4
278	ITGA4
279	ITGA5
280	ITGB3

281	ITGB6
282	PIK3R1
283	TNNT2
284	CCNA2
285	TNNI3

Table S5: Gene names, degree value, betweenness centrality (BC) and closeness centrality (CC) of 53 key targets.

BC	CC	Degree	name
8172.013685	0.002403846	148	GAPDH
7812.498333	0.002252252	122	SRC
6068.216454	0.002352941	142	TNF
5555.480588	0.002347418	140	AKT1
3012.722738	0.002178649	110	EGFR
2312.371662	0.00209205	92	PTGS2
2281.916449	0.002155172	106	BCL2
1658.902833	0.002145923	106	CASP3
1518.004547	0.00203252	84	PPARG
1400.577926	0.002061856	90	MMP9
1392.087342	0.001769912	39	DRD2
1337.491286	0.002061856	89	HSP90AA1
1259.860034	0.00204918	83	ESR1
1176.027503	0.001828154	36	ADRB2
1139.786233	0.001828154	37	ABCB1
1064.465207	0.001865672	51	PPARA
1016.243534	0.001709402	35	AGTR2
988.9605639	0.001964637	72	IL2
942.9649089	0.001858736	49	BRCA1
846.3994289	0.001821494	40	MAOA
837.4552129	0.001872659	51	REN
819.6320207	0.002008032	74	GSK3B
777.5441396	0.001792115	35	OPRM1
772.1585337	0.001766784	34	GSR
738.4783887	0.001904762	53	NR3C1
721.2399923	0.001811594	41	PKM
718.5557784	0.001897533	55	PRKACA
715.6870182	0.00177305	41	HMGCR
689.9154515	0.001919386	59	KDR
674.0024327	0.00173913	27	ITPR1
634.9693183	0.001848429	49	AR
629.9801362	0.001960784	69	SIRT1
626.6268427	0.001811594	47	MPO
625.5439886	0.001886792	53	PRKCA

619.9532039	0.001724138	27	IDO1
613.2107662	0.001930502	64	MAPK1
556.0759237	0.001798561	30	CYP19A1
538.5090981	0.001782531	37	PTGS1
530.2749737	0.001893939	53	GRB2
522.5495239	0.001841621	47	CASP8
498.9460784	0.001901141	59	EP300
480.7504364	0.001697793	27	EPHX2
479.700791	0.001757469	31	MAOB
477.0741381	0.001683502	29	ADA
476.6740333	0.001848429	51	CDK2
476.0977712	0.001890359	58	MMP2
468.4484228	0.001883239	52	HSPA5
460.4420139	0.001808318	41	PLG
454.9215281	0.001776199	28	TRPV1
449.1424182	0.001818182	43	DPP4
445.0202506	0.001760563	30	AKR1B1
419.8351525	0.001730104	34	TYMS
354.4897317	0.001776199	37	INSR

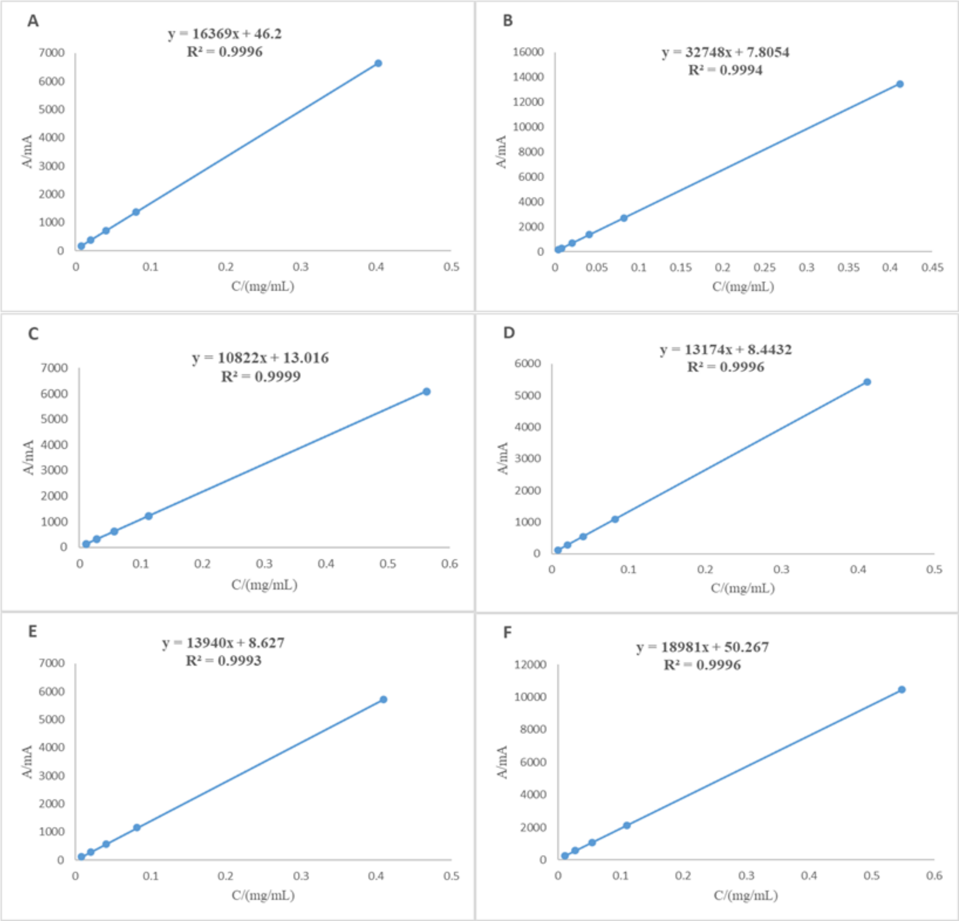

Figure S1: The calibration range and calibration curve equations for compounds; (A)

GAS, (B) HBA, (C) Parishin E, (D) Parishin B, (E) Parishin C, (F) Parishin A.

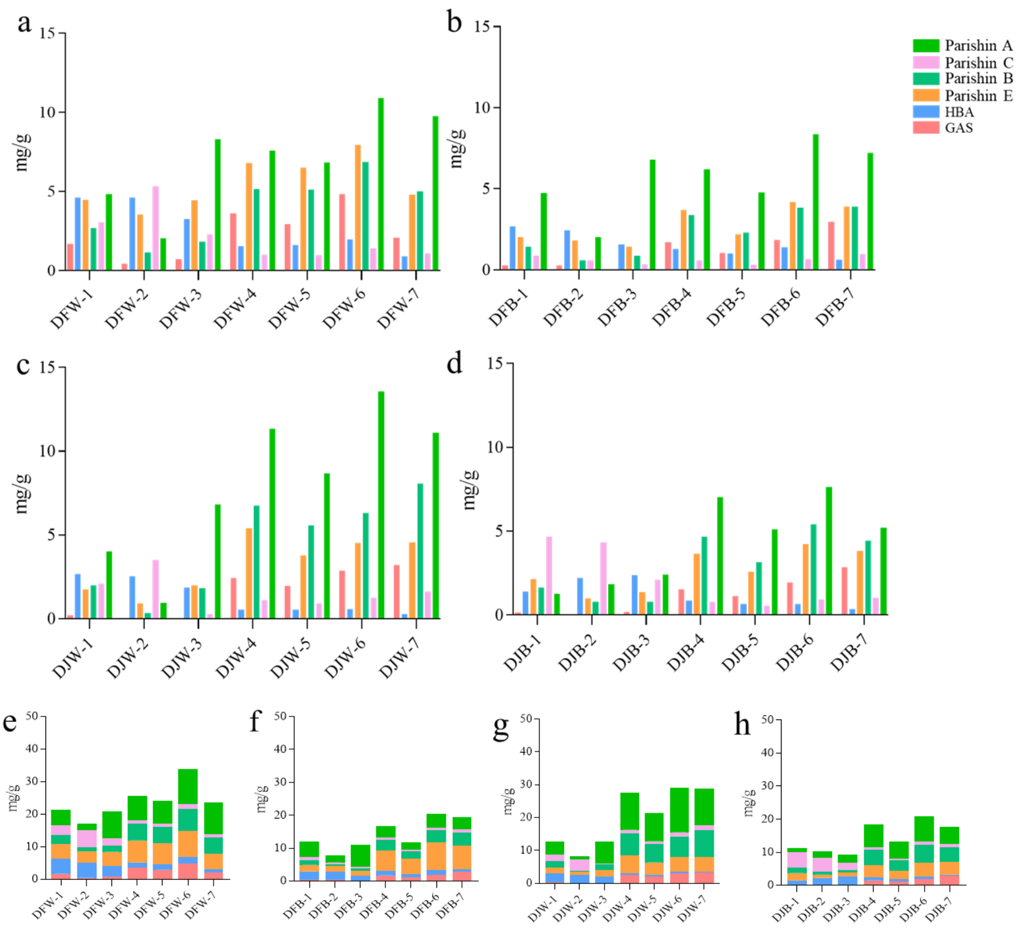

Figure S2: Difference in *Gastrodia elata* Blume composition; Difference in *Gastrodia elata* Blume composition of batches plots (a)(b)(c)(d); Histogram of the differences in GE composition among 4 groups of samples with different drying methods (e)(f)(g)(h).
